# Improving plant disease classification by adaptive minimal ensembling

**DOI:** 10.3389/frai.2022.868926

**Published:** 2022-09-08

**Authors:** Antonio Bruno, Davide Moroni, Riccardo Dainelli, Leandro Rocchi, Silvia Morelli, Emilio Ferrari, Piero Toscano, Massimo Martinelli

**Affiliations:** ^1^Institute of Information Science and Technologies, National Research Council, Pisa, Italy; ^2^Institute of BioEconomy, National Research Council, Firenze, Italy; ^3^Barilla G. e R. Fratelli S.p.A., Parma, Italy

**Keywords:** plant diseases, image classification, deep learning-artificial neural network (DL-ANN), adaptive ensemble, Convolutional Neural Networks (CNN)

## Abstract

A novel method for improving plant disease classification, a challenging and time-consuming process, is proposed. First, using as baseline EfficientNet, a recent and advanced family of architectures having an excellent accuracy/complexity trade-off, we have introduced, devised, and applied refined techniques based on transfer learning, regularization, stratification, weighted metrics, and advanced optimizers in order to achieve improved performance. Then, we go further by introducing adaptive minimal ensembling, which is a unique input to the knowledge base of the proposed solution. This represents a leap forward since it allows improving the accuracy with limited complexity using only two EfficientNet-b0 weak models, performing ensembling on feature vectors by a trainable layer instead of classic aggregation on outputs. To the best of our knowledge, such an approach to ensembling has never been used before in literature. Our method was tested on PlantVillage, a public reference dataset used for benchmarking models' performances for crop disease diagnostic, considering both its original and augmented versions. We noticeably improved the state of the art by achieving 100% accuracy in both the original and augmented datasets. Results were obtained using PyTorch to train, test, and validate the models; reproducibility is granted by providing exhaustive details, including hyperparameters used in the experimentation. A Web interface is also made publicly available to test the proposed methods.

## 1. Introduction

Early detection of plant stress is one of the most crucial practices in agriculture (Nagaraju and Chawla, 2020). Biotic stress in plants is caused by living organisms, specifically viruses, bacteria, fungi, nematodes, insects, arachnids, and weeds, while abiotic stress is caused by environmental factors such as drought, heat, cold, strong wind, flooding, and nutrient deficiencies. In agriculture, both kinds of stress are a significant cause of crop yield and quality loss leading to serious monetary harm when limits for the occurrence of the stress are exceeded (Kashef, 2020; Pantazi et al., 2020). Although over the years, genetics has made available cultivars that are increasingly resistant to various types of stress, the issue of yield and quality losses remains crucial on a global scale, especially since climate change leads to the co-occurrence of abiotic and biotic stresses (Pandey et al., 2017). Even today, the majority of the inspections are done manually by direct visual analysis, which may not make it easy to identify the disease and its type. Indeed, farmers use their naked eyes for plant inspection, which needs constant observation, high skills, and experience. Some of them are supported by guidelines with basic concepts and aiding materials (pictures/notes to identify symptoms and patterns of stress) that are relevant to distinguish between biotic and abiotic injuries and determine the possible cause and solution to adopt. At other times, farmers might require technical support to achieve a formal and complete diagnosis. In all these cases, the methodologies adopted are time-consuming and expensive (Zhang et al., 2020), often not viable for large farms or not affordable for small farms. Even the identification of weeds typology—broadleaf or grassy—is difficult in their early stages (from germination to the development of the first four/six leaves), i.e., exactly when it would be the most suitable time to counter them. This issue has increased the importance of automated infection recognition and compelled researchers to devise methods or systems that can more accurately diagnose the problem (Ma et al., 2017). In addition, the increased public concern about environmental conservation coupled with the need for more efficient agriculture necessary to cope with the simultaneous increase in population and reduction of available land) demands the introduction of new cost-effective and sustainable methods and solutions to support farmers in their daily work. In this context, machine learning techniques can finally trigger a revolution for the timely suppression of organisms harmful to plants and keep the use of chemical treatment and other forms of intervention to economically and ecologically justified levels.

Computer vision-based methods are now being considered a key enabler in this revolution. The problem has a relatively long history, including several attempts based on the use of particular imaging technologies such as thermal and stereo images (Prince et al., 2015), color and depth images (Rousseau et al., 2012), or even fluorescence imaging spectroscopy (Wetterich et al., 2013) coupled with *ad hoc* image processing pipelines. Such advanced imaging modalities might provide very specific and accurate analysis suitable for particular, especially high-revenue, crops in precision agriculture. However, standard RGB images might be preferable for the broader adoption of vision-based methods for fighting plant diseases even in low-resource and low-income areas of the world. Progresses in artificial intelligence and their excellent classification capabilities on standard images have encouraged several research lines. For instance, neural networks for plant disease classification have been used before (Huang, 2007) making use in most cases of handcrafted features and conventional computer vision pipelines. Indeed, independently of the application domain, typical computer vision techniques are composed of a pipeline of phases that almost equally contribute to the quality of the final result. In the case of image classification, in particular, the phases are (i) *preprocessing* for improving the image quality (e.g., denoising, color enhancement/balancing); *segmentation* for isolating the foreground from the background, to focus only on the useful information;*feature extraction* for obtaining only the relevant information of the foreground represented in a numeric vector (i.e., feature vector), mostly performed by a domain expert, and (iv) *classification* for learning and performing a mapping between the input feature vector and output classes.

In the last years, the paradigm shift proposed by *deep learning* (LeCun et al., 2015), consisting of a way to perform *representation learning* i.e., obtaining the data feature vector without involving a domain expert, has allowed embedding and automatically performing all the phases described above.

Convolutional Neural Networks (CNNs or ConvNets) represent Deep Learning in the scope of Computer Vision and are state-of-the-art (SOTA) in most tasks (Khan et al., 2020). Even if there are many CNN archetypes, all of them are essentially composed by stacking a variable number of modules (that usually share parameters to reduce complexity) consisting of the following layers applied sequentially:

- convolutional layers: they apply several adaptive filters to regions of the image obtaining their abstract representation;
- pooling layers: they perform aggregations which have the 2-fold effect of summarizing data, picking only relevant elements, leading to dimensionality reduction;
- non-linear activation layers: they are used to obtain a more powerful and expressive representation, reaching different levels of abstraction.

At the end of an architecture composed of the layers mentioned above, one or more fully connected layers can be stacked. This organization allows automatic preprocessing, segmentation, and feature extraction whilst classification/regression is feasible, putting a dedicated output module at the top of the architecture. The very first conceived CNN was LeNet (LeCun et al., 1989) more than 30 years ago. Again, only in the last 10 years, CNNs have been experiencing massive use and success, frequently improving the SOTA on different tasks (Krizhevsky et al., 2012; Simonyan and Zisserman, 2015; Szegedy et al., 2015; He et al., 2016; Howard et al., 2017; Hu et al., 2018; Wang et al., 2020).

Convolutional Neural Networks have been adopted to tackle the problem of plant disease classifications. For instance, Wang et al. (2017) have applied transfer learning and fine-tuning of general-purpose architectures to provide fine-grained disease severity classification in the case of the apple black rot images dataset, obtaining a best 90.16% performance using VGG16. Similarly, Ferentinos (2018) used AlexNet and GoogleNet, training the models with the use of an open database of 87,848 images, containing 25 different plants in a set of 58 distinct classes of [*plant, disease*] pairs, achieving the best performance of 99.53%. For the training and validation of deep learning paradigms, several datasets are available (Lu and Young, 2020). However, all of them have some limitations e.g., in size, variety of plants, disease coverage, and extrinsic shooting conditions (i.e., varying illumination and backgrounds). Among them, PlantVillage (Hughes and Salathe, 2015a,b) has emerged as a *de facto* open reference dataset for plant disease classification and, as such, it is considered a benchmark in this article, although it shares limitations of other datasets and, notably, the presence of standard backgrounds instead of real-world ones. It should be noticed that a large-scale benchmark dataset has been recently proposed (Liu et al., 2021), together with a new approach to disease recognition. Still, such a dataset is not freely available and has not yet gained reference value. In general, previous methods addressing the classification of PlantVillage images achieve good performance, however, they often do not sufficiently address the efficiency and complexity of the employed paradigms. Indeed, in order to achieve more significant penetration and broader adoption of the methods, the proposed paradigms should be capable of running on low resource hardware, especially on smartphones, even in the absence of remote clouds.

In view of the above consideration, in this study, we propose a new approach to plant disease classification based on adaptive minimal ensembling. The main contribution is represented by a novel approach to ensembling: different weak classifiers are trained and then combined to obtain a new combined classifier. The novelties of the proposed approach are at least 2-fold: from one side, we propose a fully trainable combination layer, granting end-to-end differentiability of the global architecture; then, in our approach, the combination layer does not act on the output layers of the weak classifiers as in other classical approaches, but the weak classifiers are truncated before. Namely, the final fully-connected layers of each weak classifier are removed, and the combination happens directly at the *deep feature* level. In addition, such an approach is brought into practice by adopting a family of SOTA models, namely EfficientNet (Tan and Le, 2019), known for their optimal complexity/performance trade-off, for each weak classifier. EfficientNet is refined by applying advanced techniques on data and processing, significantly improving the classification task. Namely, besides using ensembling, we perform transfer learning from ImageNet and introduce a novel optimizer as well as a novel validation scheme together with other minor tricks. From an experimental point of view, the article provides an advance since it shows that adaptive minimal ensembling can be used to reach top performance with a minimal computational burden compared to other promising schemes in the literature. Indeed, improving state-of-the-art, we achieve 100% accuracy on the PlantVillage dataset using an ensembling of only two weak classifiers (and thus minimal) while at the same time requiring less computational resources than the previous methodologies tested on the PlantVillage dataset. As a final contribution, carrying out the experiments both on the original PlantVillage dataset and its augmented version, we show that our method based on minimal ensembling is less sensitive to data augmentation with respect to other methods reported in the literature, in which performance significantly drops when training is not performed on the augmented dataset.

The article is organized as follows. In Section 2, we describe our designing strategy in detail (including the proposed models and the validation phases), focusing on the novelty aspects of the solution. The experimental setup is then introduced in Section 3 in which the number and typologies of experimental runs, including hyperparameters and other details, are reported in order to guarantee reproducibility. In Section 4, the obtained experimental results are reported and discussed, while Section 5 ends the article with ideas for future research.

## 2. Materials and methods

Among the pool of CNN architectures available in the SOTA for image classification, it was decided to use the EfficientNet (Tan and Le, 2019) family as the core component in this study. This was motivated by several factors.

**Figure 1:**
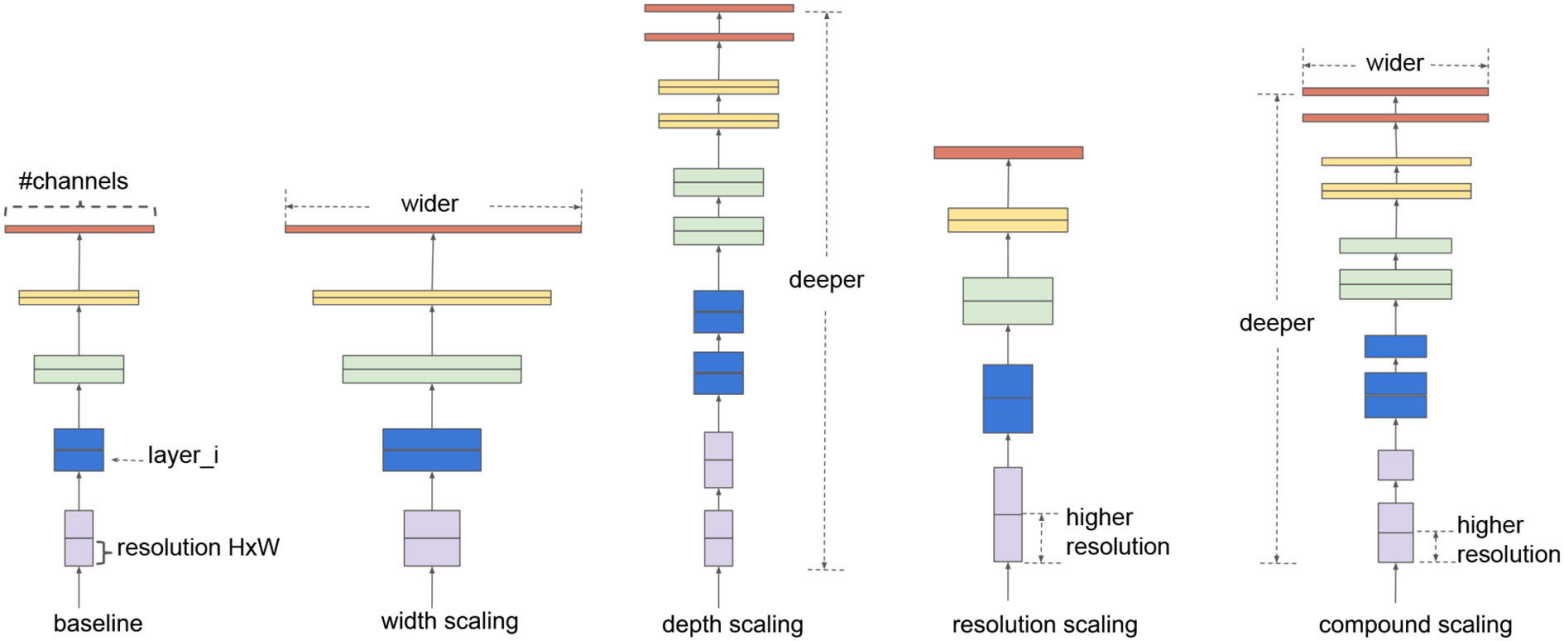
Example of scaling types, from left to right: a baseline network example, conventional scaling methods that only increase one network dimension (width, depth, and resolution), and in the end the EfficientNet compound scaling method that uniformly scales all three dimensions with a fixed ratio. Image taken from the original article (Tan and Le, 2019).

First, as the name suggests, EfficientNet improves the classification quality without having huge complexity with respect to the models having similar classification performances. EfficientNet family consists of 8 progressively improved versions (b0-b7) with limited complexity growth, all of them having the inverted bottleneck MBConv (first introduced in MobileNetV2) as the core module, which expands and compresses channels reducing the complexity of convolution. The real novelty introduced is the way EfficientNets perform scaling of the network to achieve optimal performances given a predefined complexity. In the CNN literature, there are 3 main types of scaling as shown in Figure 1:

- *depth scaling*, which consists in increasing the number of layers in the CNN; it is the most popular scaling method in the literature and allows to catch features at more levels of abstraction;
- *width scaling* means increasing the number of convolutional kernels and parameters or channels, giving the model the capability to represent different features at the same level;
- *input scaling* means increasing the size/resolution of the input images, allowing to capture more details.

Each of these scalings can be set manually or by a grid search, but there are two problems with the traditional scaling method: first, they increase the model complexity, usually exponentially, with tons of new parameters to tune and, second, after a certain level, experiments show that scaling does not improve performances. The scaling method introduced in the article is named *compound scaling* and suggests that strategically performing all scaling together delivers better results because it is observed that they are dependent. Intuitively, Tan and Le (2019) introduce a compound coefficient ϕ representing the amount of resources available to the model and find the optimal scaling combination using that amount of resources following the rules:


$$\begin{array}{l} \text{depth:}d=\alpha^{\phi }\text{ width:}w=\beta^{\phi }\text{ resolution:}r=\gamma^{\phi }\\ \text{such that }\alpha \cdot \beta^{2}\cdot \gamma^{2}\approx \text{2 and }\alpha \geq \text{1,}\beta \geq \text{1,}\gamma \geq \text{1} \end{array}$$

In this way, the total complexity of the network is approximately proportional to 2^ϕ^ (refer to the original article for more details). In the following sections, our strategy is illustrated, highlighting the differences from the previously cited works.

### 2.1. Input preprocessing

In many applications, the models are not fed directly with the images provided by the datasets, but images are preprocessed to improve the performances. In our study, since the image quality of the dataset of interest is already sufficient, we opted not to perform any image enhancement or further augmentation because an augmented version of the dataset already exists.

The only preprocessing we applied is the normalization, in order to have all data described under the same distribution (pixel values in the [0, 1] range and centered around the mean) which improves the stability and convergence of the training.

### 2.2. Transfer learning

Transfer learning (Weiss et al., 2016) is the technique of taking knowledge gained while solving one problem and applying it to a different but related problem. In this case, like most cases for image classification, the stored knowledge is brought by pre-trained models from ImageNet (Deng et al., 2009) task, since it has more than 14 million images belonging to 1,000 generic classes (including plants).

### 2.3. Avoid overfitting

In order to prevent overfitting (i.e., avoid the model being too specialized to the data from the training set with poor performances on *unknown* data), during training we use early stopping (i.e., training is interrupted after no improvements on the validation set after a certain number of epochs, called *patience*, is achieved) and regularization (i.e., adding noise to the loss, usually proportional to the norm of the model parameter vector, in order to keep parameters with low values).

### 2.4. Ensembling

**Figure 2:**
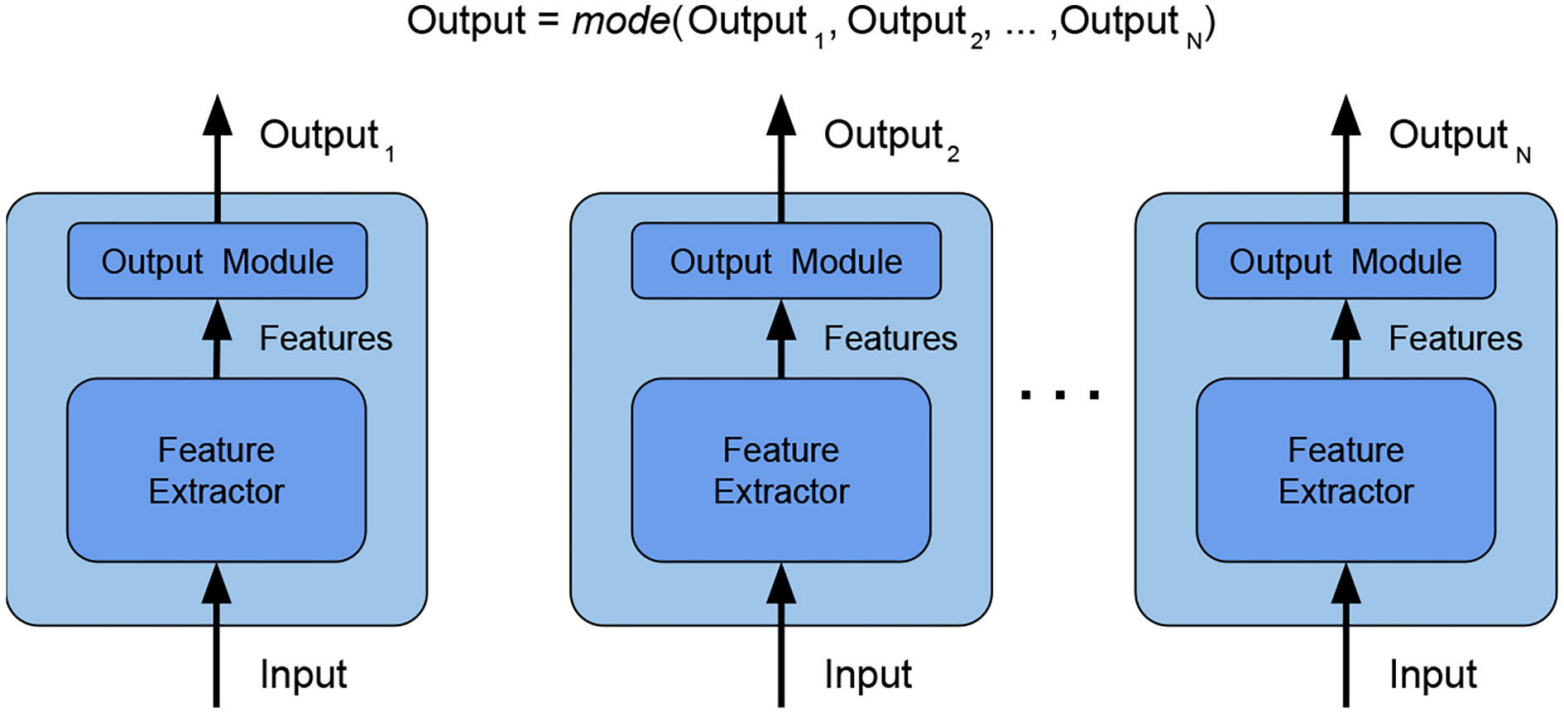
Ensemble by voting—the final label is obtained by picking the most frequent label among the weak models. In this way, the weak models are independent and the ensemble is effective with a high number of heterogeneous weak models. Weak models are CNN architectures since now represented by the sequence of Feature Extractor + Output module.

**Figure 3:**
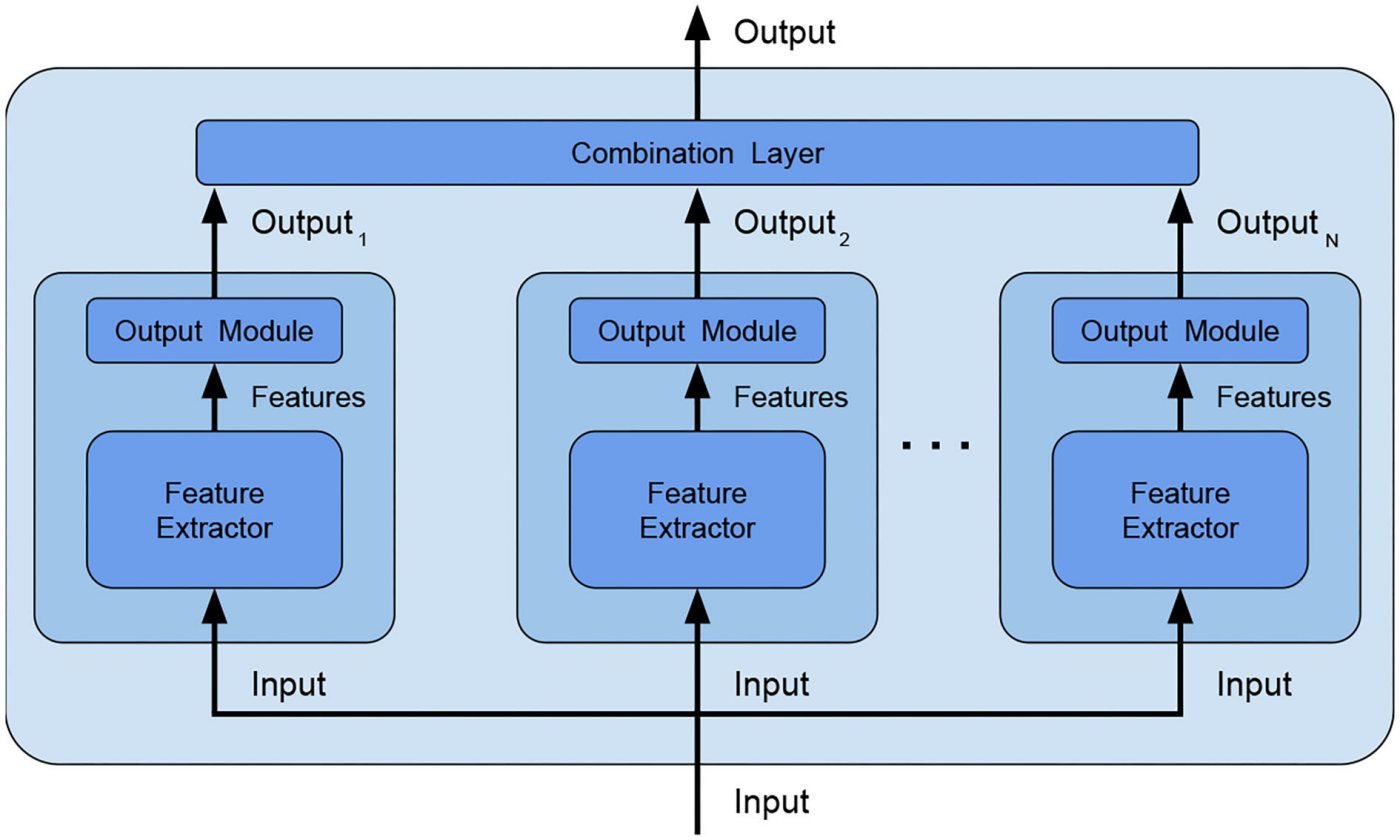
Ensemble by output combination–an additional combination layer is fed with the outputs of the weak models and combines them, in this way, the weak models are no longer independent and the combination layer can be trained to better adapt to data.

**Figure 4:**
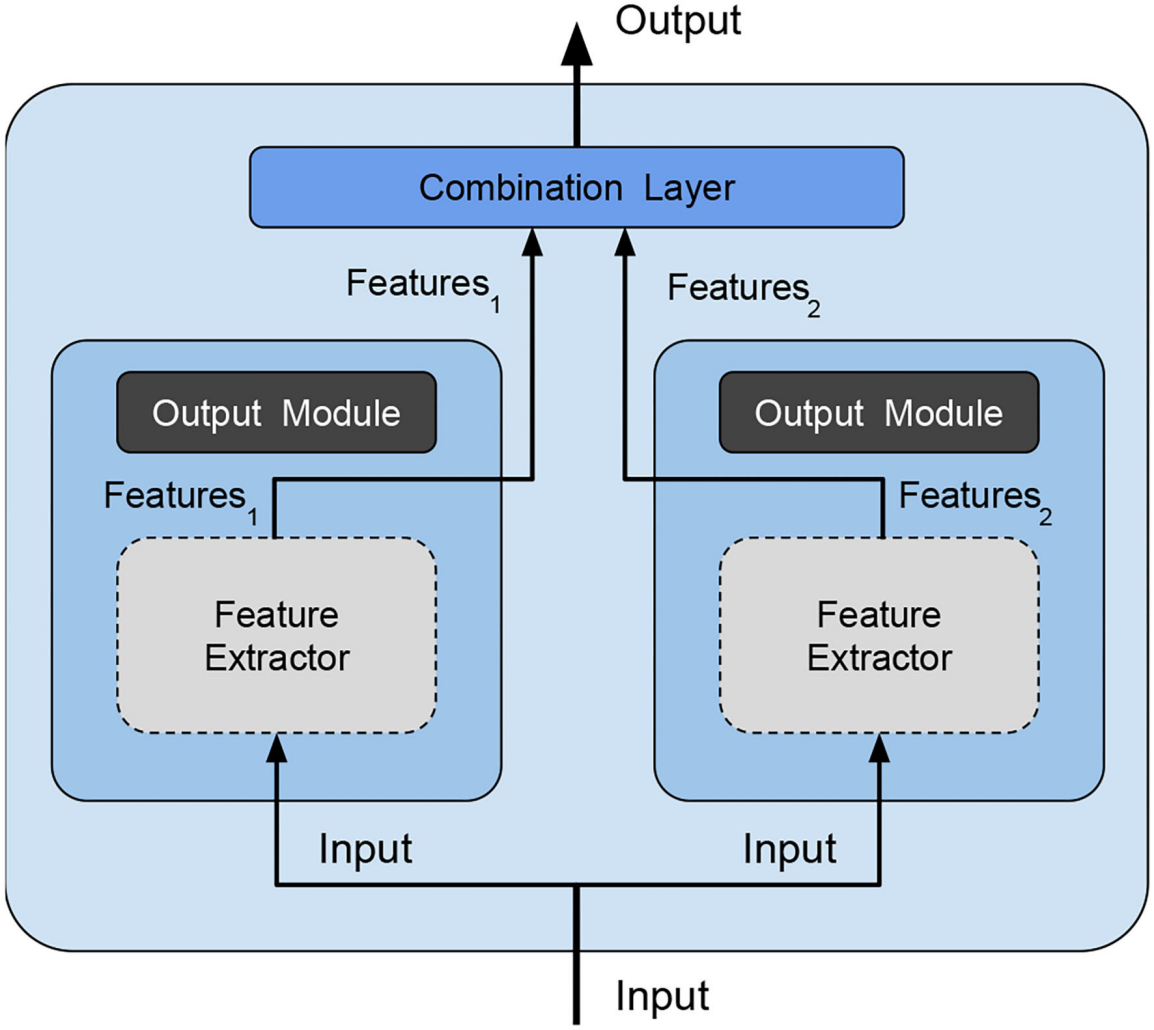
Our ensemble method—is an optimized version of the method shown in Figure 3 because we avoid redundancy and reduce complexity by deleting the output module (dark gray filled) of weak models and feeding the combination layer directly with the features extracted by each weak model. Feature extraction modules (light gray filled with dashed borders) have the parameters frozen during ensemble training.

Ensembling is the technique that combines several base models, called *weak*, in order to produce one optimal model to achieve a better performance than any of the constituent models alone (Opitz and Maclin, 1999). The studies (Sagi and Rokach, 2018; Dong et al., 2019) provide a comprehensive study on different ensembling methods supported by empirical results. Instead of performing a sort of validation to obtain the best combination of ensembling, we adopt the following heuristic choices:

- *ensemble main category*: Due to its simplicity, we decided to use *bagging*, which consists in training several independent weak models on different subsets of data. Since randomness (Ho, 1995) and heterogeneity (Gashler et al., 2008) are known to lead to good quality ensembling, subsets are picked totally random;
- *ensemble size*: The study in Bonab and Can (2016) provides the number of weak models to use for obtaining the ideal ensemble model, however, the study in Bonab and Can (2019) proves that a small number of weak models is enough to achieve high performances with low complexity. We, thus, decided to consider an ensemble size equal to 2 (i.e., the ensemble is composed of two weak models only, being therefore minimal).
- *combination type*: The typical way of combining weak models is to perform voting/averaging as shown in Figure 2 (predicting the output from all weak models and then picking the most frequent output/average of outputs), respectively for classification/regression. However, in previous study, the ensemble is only a static aggregator. In our method, we opted to perform an adaptive combination of the weak models; in addition, instead of combining the outputs (Figure 3) of weak models, the features that the CNNs extract from the input (Figure 4) are combined. In this way, the complexity of the ensemble is further reduced without diminishing its power and expressiveness. Indeed the combination layer is of the same type of the output layer as the weak models (i.e., Linear + LogSoftmax) and keeping both would introduce an unnecessary redundancy. In particular, the mechanism adopted for the fusion of the ensemble is performed first by concatenating the characteristics and then applying a linear transformation to match the output size (i.e., we perform a kind of weighted sum on the concatenated features).
- *weak models training*: Even if the study in Sollich and Krogh (1995) shows that overfitted weak models might also lead to a good adaptive ensemble, we decided to train the weak models by avoiding overfitting to save precious training time and to have weak models of higher quality.

### 2.5. Validation phases

The validation of every single model is divided into two main phases: first *end-to-end training* is performed and then followed by output module *fine-tuning*. For the first phase, transfer learning starting from the ImageNet pre-trained model is applied, introducing a new output module to adjust the output size from the 1,000 classes in the ImageNet task to the number of classes in the PlantVillage dataset. A training phase is performed using AdaBelief (Zhuang et al., 2020) optimizer which guarantees both fast convergence and generalization. The parameters used in AdaBelief are the default ones, i.e., learning rate equal to 5·10^−4^, betas (0.9, 0.999), eps 10^−16^, using weight decoupling without rectifying. After such training is concluded, the second phase starts: all the internal layers (i.e., the layers performing feature extraction) of the model obtained with the previous step are frozen, and a new training by Stochastic Gradient Descent (SDG) with a learning rate 3·10^−3^ and momentum 0.9 is performed. This leads to the fine-tuning of the output module of each classifier.

These steps conclude the validation phase for every single model. When going further to ensembling, each resulting single model is regarded as a weak model of a combined classifier and an additional dedicated pipeline is introduced for training the ensemble. First, the two best performing models are selected and truncated dropping their output module, which is replaced by a common combination layer. Then, ensemble fine-tuning (i.e., only the adaptive combination layer is trained) is performed using the same optimizer setting of the first validation phase. The reasons why we perform a dedicated pipeline for the ensemble are described in Section 3.3.

### 2.6. The plantVillage dataset

The PlantVillage dataset (Hughes and Salathe, 2015a,b) is a dataset for multiclass image classification tasks having 55,448 images (61.486 in its augmented version) divided into 39 classes representing background-only (out of domain images e.g., animals, buildings), healthy and diseased plants.

**Figure 5:**
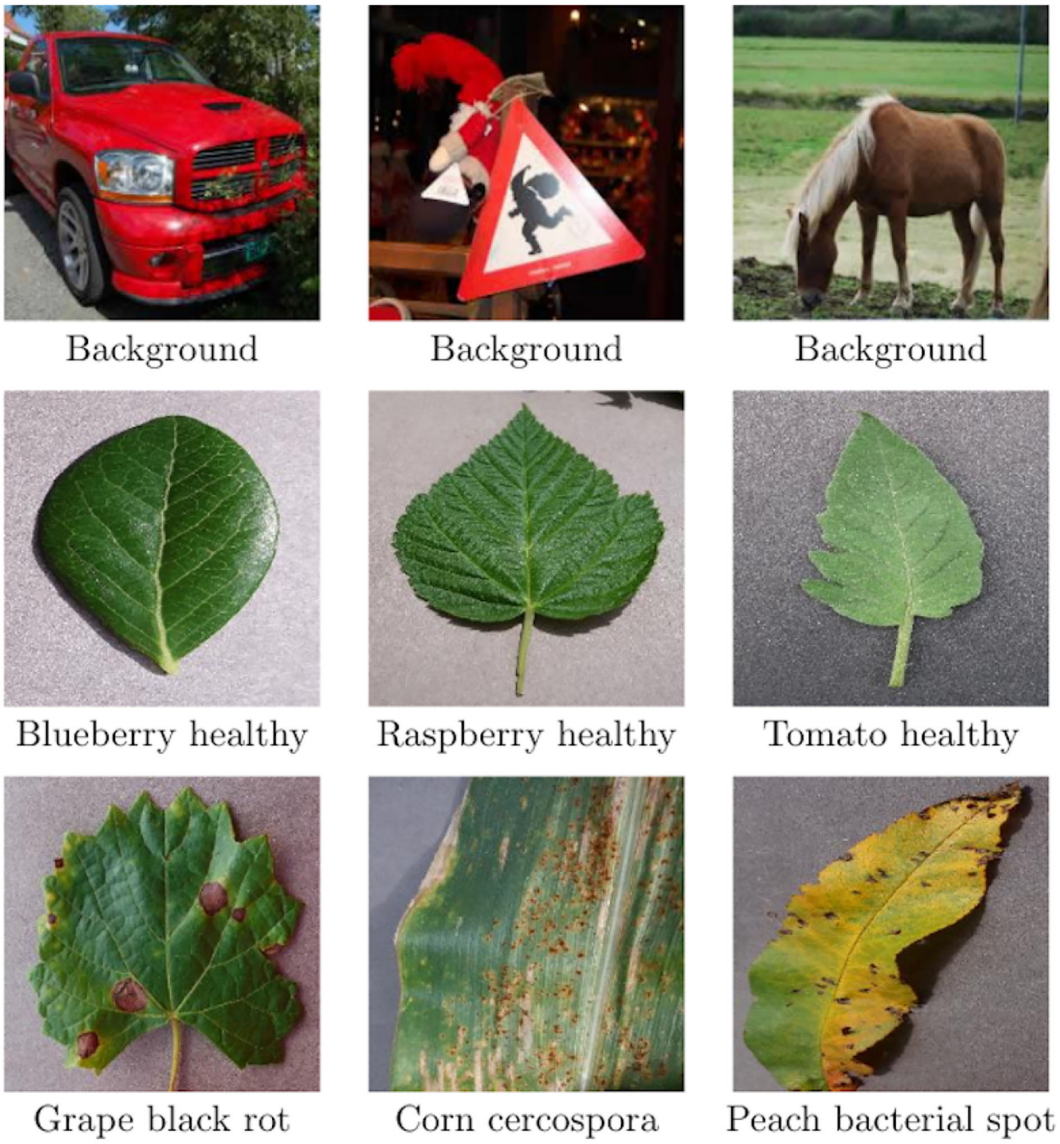
Some PlantVillage image examples. From top to bottom: 3 examples of background, 3 examples of healthy leaves, and 3 examples of diseases.

Table 1 shows that images span 14 plant species: Apple, Blueberry, Cherry, Corn, Grape, Orange, Peach, Bell Pepper, Potato, Raspberry, Soybean, Squash, Strawberry, and Tomato and contains images of 17 fungal diseases, 4 bacterial diseases, 2 mold (Oomycete) diseases, 2 viral diseases, and 1 disease caused by a mite (some examples are shown in Figure 5).

**Table 1:** Class labels distribution of PlantVillage dataset.

**Class Name**	**Class frequency**	**Class name**	**Class frequency**
Apple scab	630	Pepper healthy	1,478
Apple black rot	621	Potato early blight	1,000
Apple cedar apple rust	275	Potato healthy	1,000
Apple healthy	16,45	Potato late blight	152
Background without leaves	1,143	Raspberry healthy	371
Blueberry healthy	1,502	Soybean healthy	5,090
Cherry powdery mildew	1,052	Squash powdery mildew	1,835
Cherry healthy	854	Strawberry healthy	1,109
Corn gray leaf spot	513	Strawberry leaf scorch	456
Corn common rust	1,192	Tomato bacterial spot	2,127
Corn northern leaf blight	985	Tomato early blight	1,000
Corn healthy	1,162	Tomato healthy	1,591
Grape black rot	1,180	Tomato late blight	1,909
Grape black measles	1,383	Tomato leaf mold	952
Grape leaf blight	985	Tomato septoria leaf spot	1,771
Grape healthy	1,162	Tomato spider mites	1,676
Orange haunglongbing	5,507	Tomato target spot	1,404
Peach bacterial spot	2,297	Tomato mosaic virus	373
Peach healthy	360	Tomato yellow leaf curl virus	5,357
Pepper bacterial spot	997		

Some diseases are typical of particular plant phenotypes, there are also healthy leaf and background-only images. The frequency values refer to the standard dataset, while in the case of its augmented version only the classes with a size less than 1,000 were augmented to reach 1,000 images (classes having more images are not modified).

## 3. Experimental setup

In this section, we describe the design choices justified by prior observations. All the reported experimental results were obtained using the PyTorch (Paszke et al., 2019) open-source machine learning framework.

A somewhat non-conventional training/validation/test splitting has been used in the experiments to reproduce the conditions closest to the study in Ümit Atila et al. (2021) representing the SOTA for PlantVillage while doing this work. More in detail, datasets have been split into training (90%), validation (7%), and test (3%). While obviously splits have the same sizes as previous articles, however, since the picks are random, the actual elements in each subset may vary. Moreover, we performed stratification (i.e., preserving the classes ratio). Besides the non-conventional split, in the result and discussion section, a classic split is also considered to show the suitability of our strategy also in this case.

### 3.1. Loss and metrics

**Training Loss:** Due to the multiclass nature of the problem, the Cross-Entropy Loss (which exponentially penalizes differences between predicted and true values, expressed as the probability of class belonging) is used. For this reason, the model output has a size of 39 (i.e., number of classes), and each element output[*i*] represents the probability that the input model belongs to class *i*.

**Validation and test metrics:** For the validation set evaluation, we decided to use the Weighted F1-score because this takes into account both correct and wrong predictions (true/false positive/negative) and weighting allows us to manage any imbalance of the classes (more representative classes have a greater contribution). On the other hand, in order to compare our results with previous studies, we used accuracy to evaluate the test set.

### 3.2. Hyperparameters

In order to save time, after an initial coarse search, we fixed some hyperparameters:

- Early-stopping patience set at 10: Because deep models have relatively fast convergence and they usually start overfitting after convergence, so there is no need to have much patience;
- Batch size set at 32: Because it is the maximum size allowed on the GPUs we used to perform model training and lower values showed no improvement (and would make training slower). Stratification even inside the batches would have been desirable but this is possible only if the batch size is greater than the number of the classes, which is not our case;
- Input image size fixed to 256: In order to preserve input image quality, a larger size would ruin the images, the lower size would reduce details;
- Mean and SD used for normalization:

$$\begin{array}{l} \;\mu =[0.4683,0.5414,0.4477]\;\sigma =[0.2327,0.2407,\\ 0.2521]\text{ for augmented dataset}\\ \;\mu =[0.4685,0.5424,0.4491]\;\sigma =[0.2337,0.2420,\\ 0.2531]\text{ for original dataset} \end{array}$$

Moreover, we observed that regularization was not needed during the end-to-end training phase, and, in some cases, it even led to worse results. Regularization is then used only in weak models (not used for ensemble) fine-tuning step, with the following hyperparameters:

- regularization type: Lasso (L1), Ridge (L2)
- regularization factor (λ): 0, 10^−4^, 5·10^−4^, 10^−3^.

For each combination, we used 3 different random seeds in order to obtain different model parameter initialization values, and different train/valid/test splits (useful to obtain random heterogeneous weak models for ensemble).

Considering each dataset, we had the following combinations:

- End-to-end phase: 8 (EfficientNet architectures ranging from b0 to b7) × 3 (random seeds) = 24 combinations;
- Fine-tuning phase: 24 (end-to-end phase results) × 2 (regularization types) × 4 (regularization factors) = 192 combinations;

Since there are two datasets (standard, augmented), the total number of runs is equal to 2 × (192) = 384, excluding ensembling which is addressed in Section 3.3 below.

### 3.3. Adaptive minimal ensembling, improving performances with minimum complexity

The last experimental step is to evaluate the performance of ensembling. We opted to perform this step using only EfficientNet-b0 as weak models and not the full family of weak models trained in Section 3.2. This choice was motivated by two observations: first of all, the performances of all EfficientNet variants after the end-to-end phase are very similar as we show in Table 2, but the b0 variant is much simpler (≈ 5M parameters vs ≈ 66.7M parameters of b7), so we decided to investigate on the simplest type of ensemble, even in terms of the number of parameters (and not just in terms of ensemble size using only 2 weak models). Moreover, we skipped the fine-tuning phase as it is used to optimize those parameters that are removed during ensembling because they are redundant (notice, however, that we performed fine-tuning anyway before, in order to collect experimental results to allow fair comparison and show the improvements ensembling can offer over single models). For the ensemble phase, we followed another validation scheme that is, for each version of the dataset, the following:

1. Five end-to-end training of EfficientNet-b0 with different initializations and data splits results in Table 3;
2. No fine-tuning because the parameters involved in this phase would be removed during ensemble;
3. Five fine-tuning of a minimal ensemble composed of the two best weak models obtained at point 1, using different initializations and data splits.

**Table 2:** Table with best Weighted F1-score results, for each EfficientNet variant, after the first phase of validation (i.e., end-to-end training).

	**Original**			**Augmented**		
**Model**	**Test**	**Valid**	**Train**	**Test**	**Valid**	**Train**
EfficientNet-b0	99.6995	99.8454	99.9960	99.7832	99.8374	99.9982
EfficientNet-b1	99.5793	99.8454	100.000	99.8916	99.8141	99.9928
EfficientNet-b2	99.5192	99.7681	99.9140	99.7832	99.8374	99.9982
EfficientNet-b3	99.6394	99.8712	99.9860	99.8374	99.9303	99.9964
EfficientNet-b4	99.6995	99.8454	99.9980	99.5664	99.8606	99.9982
EfficientNet-b5	99.7596	99.7939	99.9920	99.9458	99.8606	99.9982
EfficientNet-b6	**99.7596**	**99.8712**	**99.9880**	99.7290	99.8141	99.9675
EfficientNet-b7	99.5192	99.8454	99.9960	**99.8916**	**99.9303**	**99.9982**

The values of the best architectures are in bold.

**Table 3:** Table with Weighted F1-score of the models (best to worst) of the 5 end-to-end training runs using EfficientNet-b0 variants only that will be the weak models of the simplest ensemble.

	**Original**			**Augmented**		
**Model**	**Test**	**valid**	**Train**	**Test**	**valid**	**Train**
EfficientNet-b0	99.8197	99.9485	100.000	99.7832	100.000	100.000
EfficientNet-b0	99.8197	99.8969	100.000	99.6748	99.8838	100.000
EfficientNet-b0	99.7596	99.8454	100.000	99.8374	99.8374	99.9980
EfficientNet-b0	99.7596	99.7423	100.000	99.8374	99.6515	100.000
EfficientNet-b0	99.5793	99.9227	99.9960	99.7832	99.6747	100.000

In this way, only 10 runs per dataset are performed, which are drastically fewer than 192 as described in Section 3.2 and every run is much faster. All training runs had the same configuration: AdaBelief optimizers with a learning rate 5·10^−4^, betas (0.9, 0.999), eps 10^−16^, using weight decoupling without rectifying, and Weighted F1-score as validation metric.

It must be noted that using different seeds for each validation phase, both for end-to-end and for ensemble fine-tuning, produces different dataset splits: this can be, therefore, viewed as cross-validation and, by averaging the values in Table 4, we obtain the same results proving that our solution is consistent.

**Table 4:** Table with the Weighted F1-score of the 5 ensemble runs (best to worst) composed of the two best EfficientNet-b0 variants.

	**Original**			**Augmented**		
**Model**	**Test**	**valid**	**Train**	**Test**	**valid**	**Train**
EfficientNet-b0 ensemble	100.000	100.000	100.000	100.000	100.000	100.000
EfficientNet-b0 ensemble	100.000	100.000	100.000	100.000	100.000	100.000
EfficientNet-b0 ensemble	100.000	100.000	100.000	100.000	100.000	100.000
EfficientNet-b0 ensemble	100.000	100.000	100.000	100.000	100.000	100.000
EfficientNet-b0 ensemble	100.000	100.000	99.9980	100.000	99.9768	100.000

## 4. Results and discussion

Every validation step results in incremental improvements: We discuss them one by one in the following.

**End-to-end phase:** as shown in Table 2 results are very similar, moreover considering both datasets there is no architecture being always the best/worst in both cases. It is also important to say that already in this phase we improved the SOTA: indeed, the best results until this study were obtained by Ümit Atila et al. (2021) with a Validation Accuracy of 97.62% and Test Accuracy of 98.31% for the standard dataset, Validation Accuracy of 98.97% and Test Accuracy of 99.38% for the augmented dataset. We relate this improvement to our design choices: AdaBelief optimizer, performing stratification during dataset, using Weighted F1-score as validation metric, and using normalization parameters taken from datasets instead of ImageNet defaults.

**Fine-tuning phase:** As shown in Table 5, EfficientNet-b7 is the best architecture in both datasets. In major cases, this phase led to no improvements, in a few cases, it improves performances on training data without getting worse on validation/test (i.e., improvements without overfitting). The improvements are more noticeable for the standard dataset (because in the first phase, results were lower), especially for the b7 variant obtaining improvements overall. We observed no differences among different regularization types and factors, but it was needed (because with λ = 0, we got no improvements).

**Table 5:** Table with best Weighted F1-score results, for each EfficientNet variant, after the second phase of validation (i.e., end-to-end training + fine-tuning).

	**Original**			**Augmented**		
**Model**	**Test**	**valid**	**Train**	**Test**	**valid**	**Train**
EfficientNet-b0	99.6995	99.8454	100.000	99.8916	99.8838	100.000
EfficientNet-b1	99.6995	99.8969	100.000	99.8916	99.8374	99.9982
EfficientNet-b2	99.5793	99.8712	100.000	99.7832	99.9303	99.9982
EfficientNet-b3	99.7596	99.8712	99.9900	99.8374	99.9303	99.9964
EfficientNet-b4	99.6995	99.8454	99.9980	99.5664	99.8606	99.9982
EfficientNet-b5	99.7596	99.7939	99.9920	99.9458	99.8606	99.9982
EfficientNet-b6	99.8197	99.8712	99.9900	99.7290	99.8141	99.9675
EfficientNet-b7	**99.8197**	**99.8712**	**100.000**	**99.8916**	**99.9303**	**100.000**

The values of the best architectures are in bold.

**Ensemble:** This phase gave a huge peak of improvement in both datasets, obtaining a perfect 100% accuracy on both versions of the dataset (Table 4) being much less complex (10M vs. 66.7M total parameters of EfficientNet-b7 as shown in Table 6).

**Table 6:** Table comparing complexity (measured as the number of parameters) of the SOTA models on PlantVillage task.

**Model**	**Dataset**	
	**Original**	**Augmented**
Mohanty et al. (2016) (GoogleNet)	≈ 7M	-
Too et al. (2019) (DenseNets-121)	≈ 7.9M	-
Chen et al. (2020) (MobileNet-Beta)	≈ 3.7M	-
Ümit Atila et al. (2021) (EfficientNet)	≈ 30.5M	≈ 19.5M
End-to-end (ours)	≈ 43.2M	≈ 66.7M
Fine-tuning (ours)	≈ 66.7M (100k)	≈ 66.7M (100k)
Minimal ensemble (ours)	≈ 10M (100k)	≈ 10M (100k)

The minimal ensemble is the least complex because even if it has 10M parameters it can be considered as 5M because the weak models are independent and can be executed in parallel. Moreover, during training, only the 100k parameters of the combination layers are trained.

We finally summarize the design choices and the improvements they led to.

**Transfer learning:** Helped to speed up and optimize (because training from scratch done in the preliminary analysis always led to poor results) the end-to-end training phase;

**Adabelief optimizer:** Allowed to reach lower minimal points due to its high convergence speed without losing generalization power (previous SOTA work used Adam);

**Stratification and weighted F1-score:** Reduced the problems due to high data imbalance, indeed in the augmented dataset, there is less imbalance and with the same condition, there are better performances on it (previous SOTA work used normal accuracy);

**Regularization:** Harmful during end-to-end training but essential during fine-tuning, even if there is no seeming difference among regularization types or factors (previous SOTA work does not seem to use regularization);

**Ensembling:** Using two weak models is enough to have meaningful improvements if the models are heterogeneous enough (i.e., trained on different subsets of data) even if they are very simple. This avoids overfitting since the training of weak models increased the base quality and reduced overall execution time. Finally, performing ensembling on features instead of outputs further reduced the complexity and deleted redundancies.

Now, we consider the comparison between our solution and the models representing the SOTA on the PlantVillage task over the years: Tables 6, 7 show that our design choices, different from previous studies (i.e., AdeBelief optimizer, stratification, weighted-F1, regularization) improved performances if we consider single models. Our minimal ensemble method introduced in this study had a 2-fold improvement: perfect accuracy score without increasing model complexity. Moreover, the feature extractor modules are frozen making the real trainable parameters number very low even in the ensemble (100k trainable parameters over the 10M in total), and the execution of weak models can be performed in parallel since they are independent (thus, the execution time for a 10M parameters ensemble is close to the execution time of a single 5M parameters model).

**Table 7:** Table comparing accuracies (measured as correct prediction over the whole dataset) of the SOTA models on the PlantVillage task.

**Model**	**Dataset**	
	**Original**	**Augmented**
Mohanty et al. (2016) (GoogleNet)	99.3500%	-
Too et al. (2019) (DenseNets-121)	99.7500%	-
Chen et al. (2020) (MobileNet-Beta)	99.8500%	-
Ümit Atila et al. (2021) (EfficientNet)	99.9100%	99.9700%
End-to-end (ours)	99.9729%	99.9904%
Fine-tuning (ours)	99.9856%	99.9919%
Minimal ensemble (ours)	100.000%	100.000%

Since the end-to-end phase, our study was shown to improve the SOTA.

As said before, we performed a split to make a fair comparison with the SOTA (i.e., 90% train, 7% validation, and 3% test). However, to prove its robustness, our solution has also been tested using a traditional 80/10/10 split: Few weak models were trained and then two best were used to run some ensemble fine-tuning, for each dataset version. The results in Table 8 prove that our solution is suitable also for traditional data splits.

**Table 8:** Table showing the accuracies of the two weak models and the fine-tuning ensembling them, using a traditional 80/10/10 split.

	**Original**			**Augmented**		
**Model**	**Test**	**valid**	**Train**	**Test**	**valid**	**Train**
EfficientNet-b0 weak1	99.8738	99.8557	100.000	99.8536	99.8536	100.000
EfficientNet-b0 weak2	99.8377	99.9278	100.000	99.7886	99.9187	100.000
Ensemble (weak1 + weak2)	100.000	100.000	99.9864	100.000	100.000	100.000

A web application was also implemented to show the results, allowing to pick an image from the datasets and showing its classification and probabilities; this is publicly accessible at the following address: http://plantvillage.isti.cnr.it:9090.

## 5. Conclusion

Identifying plant diseases and devising optimal adaptive countermeasures can bring significant improvement in crop quality and yields. In this context, expert systems based on artificial intelligence can be a valid aid to farmers, yet there are still no operational services for most crops. This article contributed to the creation of artificial intelligence modules for plant disease classification with high accuracy and efficiency. Indeed, it has been described how specific target design choices can lead to a relevant performance improvement over an already top-rated solution without efficiency loss. The first improvement of the state of the art was reached by using a different optimizer (i.e., Adabelief) in combination with techniques to deal with unbalanced data (i.e., stratification and Weighted F1-score). A family of classifiers based on the EfficientNet architecture has been proposed with similar accuracy but increasing complexity.

The second gain in performance was obtained by introducing a minimal adaptive ensemble model using the combination of the features of the two least complex weak models: while the number of total parameters doubled with respect to the least complex model, perfect accuracy was achieved. To the best of our knowledge, none of the above-mentioned techniques have been ever used before. Doubling the number of total parameters, however, did not increase the total complexity for two reasons: First, only a tiny part of parameters is trained during the ensemble training step (100k over 10M), and, second, the weak models can process input in parallel (therefore, the overall execution time is very close to the execution time of a single weak model). In addition, by minimal ensembling, the performance gap between original and augmented datasets is reduced; it could be argued that this type of ensembling can be helpful in cases where data balancing and augmentation are not feasible or not convenient in terms of computational time/resources. These perspectives will be studied in the future, considering other disparate domains and reference benchmarking datasets. In particular, we are currently investigating the use of adaptive minimal ensembling and the gains it is possible to achieve both in absolute average precision and in the ratio between precision and complexity, toward more sustainable use of artificial intelligence.

Regarding the precision agriculture domain, having achieved top performance on the *de facto* benchmarking dataset, the research will pursue the possibility to provide operational services to farmers to identify and recognize plant diseases. To this end, a participatory approach is being followed to gather a large dataset in the specific domain of durum wheat crop culture from pictures taken in the field by farmers, also using mobile devices. This initiative is leading to a realistic and more complex dataset to champion the methods proposed in this article.

## Data availability statement

The original contributions presented in the study are included in the article/supplementary material, further inquiries can be directed to the corresponding author.

## Author contributions

All the authors contributed to this article that was coordinated by PT and MM. The data analysis and the experiments were done and manuscript was written by AB, DM, and MM. The article drafting was done by AB, DM, PT, and MM. All the authors revised the manuscript several times and approved the article.

## Funding

This study was partially supported by the *Barilla Agrosatplus* research project DBA.AD001.034.

## Conflict of interest

Authors SM and EF are employed by Barilla G. e R. Fratelli S.p.A. The remaining authors declare that the research was conducted in the absence of any commercial or financial relationships that could be construed as a potential conflict of interest.

## Publisher's note

All claims expressed in this article are solely those of the authors and do not necessarily represent those of their affiliated organizations, or those of the publisher, the editors and the reviewers. Any product that may be evaluated in this article, or claim that may be made by its manufacturer, is not guaranteed or endorsed by the publisher.
